# Meta-analysis of the clinical characteristics and prognostic relevance of NOTCH1 and FBXW7 mutation in T-cell acute lymphoblastic leukemia

**DOI:** 10.18632/oncotarget.18576

**Published:** 2017-06-19

**Authors:** Rong-Bin Liu, Jian-Gui Guo, Tian-Ze Liu, Cheng-Cheng Guo, Xin-Xiang Fan, Xiao Zhang, Wei-Han Hu, Xiu-Yu Cai

**Affiliations:** ^1^ State Key Laboratory of Oncology in South China, Guangzhou, China; ^2^ Collaborative Innovation Center for Cancer Medicine, Guangzhou, China; ^3^ Department of Molecular Diagnostics, Sun Yat-Sen University Cancer Center, Guangzhou, China; ^4^ Department of Radiation Oncology, Sun Yat-Sen University Cancer Center, Guangzhou, China; ^5^ Department of Neurosurgery, Sun Yat-Sen University Cancer Center, Guangzhou, China; ^6^ Department of Urology, Sun Yat-Sen Memorial Hospital, Guangzhou, China; ^7^ Department of VIP Region, Sun Yat-Sen University Cancer Center, Guangzhou, China; ^8^ Department of Radiation Oncology, The First People's Hospital of Foshan, Foshan, China

**Keywords:** T-ALL, NOTCH1, FBXW7, prognosis, meta-analysis

## Abstract

The NOTCH1 signaling pathway is crucial for T-cell development, and NOTCH1 and/or FBXW7 mutations are frequently detected in T-cell acute lymphoblastic leukemia (T-ALL). We performed a systematic review and meta-analysis of 18 randomized controlled trials (RCTs) to assess the prognostic impact of mutations in the NOTCH1 pathway. After retrieving relevant articles from PubMed, EMBASE, and the Cochrane Library, we investigated overall survival (OS) and event-free survival (EFS) with hazard ratios (HRs) using fixed-effects or random-effects models and conducted subgroup analyses based on population and mutation status. NOTCH1/FBXW7 mutations correlated significantly with better prognosis (5-year EFS: HR, 0.57; 95% confidence interval [CI], 0.46 to 0.68; *P* < 0.001 and 5-year OS: HR, 0.61; 95% CI, 0.51 to 0.74; *P* < 0.001). The HR for 5-year EFS and OS with NOTCH1 mutations were 0.63 (95% CI, 0.53 to 0.75) and 0.76 (95% CI, 0.60 to 0.95), respectively; with FBXW7 mutations, they were 0.82 (95% CI, 0.60 to 1.11) and 0.79 (95% CI, 0.55 to 1.12), respectively. However, differences between children and adults showed no significance. We conclude that the presence of NOTCH1/FBXW7 mutations is an independent prognostic factor for 5-year EFS and 5-year OS.

## INTRODUCTION

T-cell acute lymphoblastic leukemia (T-ALL) accounts for approximately 15% and 25% of ALL in pediatric and adult patients, respectively [1], and is clinically characterized as a high-risk malignancy with a relapse rate of approximately 30% [2]. This disease is commonly correlated with acquired chromosomal translocations and other genetic or epigenetic abnormalities, leading to aberrant expression of a series of transcription factors [3]. Most of the chromosome rearrangements involve NOTCH1, which is constitutively active because of t (7;9) (q34:q34.3) activating mutations, also known as Notch homolog 1 and translocation-associated Drosophila, human genes encoding single-pass transmembrane receptor. Mutations in the NOTCH1 gene are the most common genetic abnormalities found in T-ALL, affecting more than 50% of patients [4, 5]. Mutations affect two domains—the extracellular heterodimerization domain (HD), where mutations lead to ligand-independent cleavage, and the C-terminal proline, glutamic acid, serine, and threonine (PEST)–rich domain truncating mutations [6]. Weng et al. [3] demonstrated activation of NOTCH1 mutations in more than 50% of pediatric T-ALL patients. Aberrant NOTCH1 signaling was originally linked to the pathogenesis of T-ALL by the cloning of the t (7;9) (q34; q34.3) chromosomal translocation, which leads to the expression of a truncated and constitutively active form of NOTCH1. The γ-secretase inhibitors (GSIs), which block the proteolytic cleavage of the NOTCH receptors and suppress the release of activated NOTCH1 (ICN1) from the membrane, have been proposed as a potential therapy in T-ALL. Moreover, F-box and WD-40 domain protein 7 (FBXW7) promotes proteasome degradation in activated NOTCH1. FBXW7 is an E3 ubiquitin ligase that recognizes the PEST domain of ICN1 and accelerates the termination of NOTCH1 signaling in the nucleus [7]. Hence, NOTCH1 mutations, FBXW7 mutations, or both lead to activation of the NOTCH1 pathway [8]. Studies have examined the correlation between NOTCH1/FBXW7 status and T-ALL. However, the outcome and results are inconsistent [9–19], and the impact of NOTCH1/FBXW7 mutations on long-term outcome in T-ALL patients is still undetermined [20].

In this meta-analysis, we investigated the relevance of NOTCH1 mutations, FBXW7 mutations, or both in relation to long-term prognosis.

## RESULTS

### Study characteristics

Trials were classified into three subgroups, NOTCH1 alone, FBXW7 alone, and NOTCH1/FBXW7, because NOTCH1 and FBXW7 belong to the same signaling pathway. In each group, children and adults were analyzed. Patients younger than 18 years were assigned to the child group. Patients older than 18 years were assigned to the adult group. Overall survival (OS) was defined as the time from diagnosis to death by any cause or to last follow-up. Event-free survival (EFS) was defined as the time from diagnosis to the date of last follow-up in complete remission or first event. Events were resistance to therapy (non-response), relapse, secondary neoplasm or death by any cause. Failure to achieve remission because of early death or non-response was considered an event at time zero.

### NOTCH1 and FBXW7 mutations

A total of 866 patients were screened for both NOTCH1 and FBXW7 mutations in 12 trials [9–19, 21]. Details are shown in Table 1. Based on age, all patients were divided into an adult subgroup and a child subgroup.

**Table 1:** Summary of 12 trials of NOTCH1/FBXW7 mutations included in this meta-analysis

Source [Reference]	Country	Age	Protocols/Trials	Study groups	No. of patients	No. of events	
						**OS**	**EFS**
Bonn et al (2013)	USA	Child	NHL-BFM	Mut	74	na	62
				WT	42	na	28
Clappier et al (2010)	Finland	Child	EORTC58951	Mut	80	31	26
				WT	54	18	16
Erbilgin et al (2010)	Turkey	Child	ALL-BFM protocols	Mut	20	15	16
				WT	67	39	54
Fogelstrand et al (2014)	Sweden	Child	NOPHO ALL-1992 and ALL-2000	Mut	47	22	28
				WT	32	17	14
Jenkinson et al (2013)	UK	Child	MRC UKALL 2003	Mut	101	37	36
				WT	57	16	15
Myoung-Ja et al (2008)	Japan	Child	(JACLS) protocols ALL-97	Mut	22	22	21
				WT	33	26	23
Abdelali et al (2010)	France	Adult	LALA-94 and GRAALL	Mut	159	25	21
				WT	73	4	4
Baldus et al (2009)	Germany	Adult	GMALL 05/93 and 06/99	Mut	67	na	28
				WT	33	na	9
Mansour et al (2009)	UK	Adult	UKALLXII/ECOGE2993 protocol	Mut	58	11	11
				WT	30	4	3
Mansur et al (2012)	Brazil	Adult	BFM protocols	Mut	62	36	25
				WT	48	23	23
Trinquand et al (2013)	France	Adult	GRAALL-2003; GRAALL-2005	Mut	143	39	34
				WT	69	8	7
Vlierberghe et al (2013)	USA	Adult	E2993 ECOG	Mut	33	19	na
				WT	20	5	na

Abbreviations: WT, wild type; Mut, mutational status; OS: overall survival at 5 years; PFS: progression-free survival at 5 years. NA indicates not available.

### 5-year overall survival

**Figure 1:**
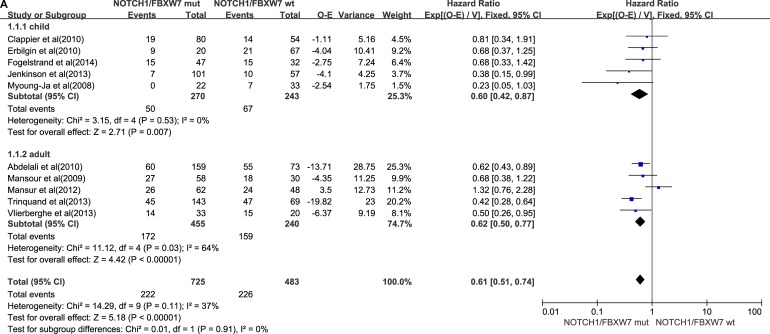

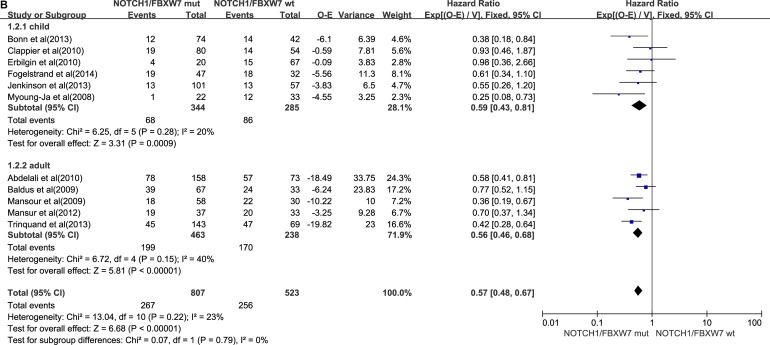
NOTCH1/FBXW7 mutations group (**A**) Forest plot of hazard ratio (HR) of 5-year overall survival. (**B**) Forest plot of hazard ratio (HR) of 5-year progression-free survival. The estimation of the HR of each individual trial corresponds to the middle of squares and the horizontal line gives 95% confidence interval. The closed diamond shows overall HR with its 95% CI. HR < 1 and 95% CI, excluding 1 indicate improved survival for the NOTCH1/FBXW7 mutations status arm compared with the wild-type NOTCH1/FBXW7 arm. O–E, observed minus estimated number of death. V, variance.

We analyzed the 5-year OS of all patients and found that two trials did not include this value. We eliminated those trials when performing the analysis. The pooled HR from the remaining trials was 0.61 (95% CI, 0.51–0.74; *P* < 0.00001). HR of the child subgroup was 0.60 (95% CI, 0.42–0.87; *P* = 0.007) . HR of the adult subgroup was 0.62 (95% CI,0.50–0.77; *P* < 0.00001). As the high degree of heterogeneity was performed, the HR of the adult subgroup was not sufficient (Figure 1A). We concluded that NOTCH1 and FBXW7 mutations were correlated with a better survival in T-ALL pediatric patients.

### 5-year event-free survival

One adult study did not include the relevant statistics for 5-year EFS. We eliminated that study. The pooled HR of the remaining studies was 0.57 (95% CI, 0.48–0.67; *P* < 0.00001). In the child subgroup, HR was 0.59 (95% CI, 0.43–0.81; *P* = 0.00009). In the adult subgroup, HR was 0.56 (95% CI, 0.46–0.68; *P* < 0.00001). Figure 1B shows that T-ALL patients who harbored NOTCH1 and FBXW7 mutations had a better prognosis than those who did not. Mutations in the child subgroup were not significantly different from those in the adult subgroup.

### NOTCH1 mutation

**Figure 2:**
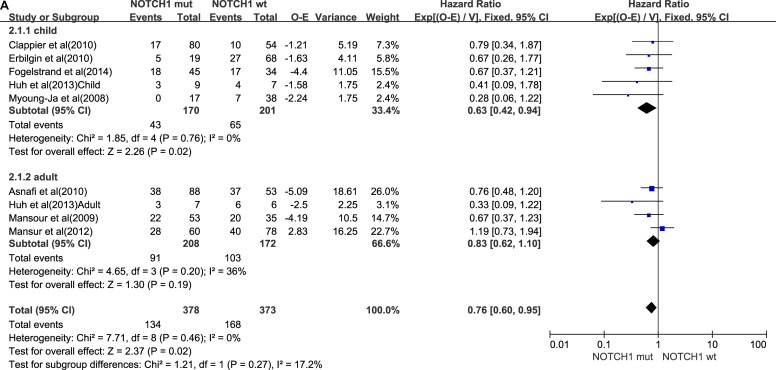

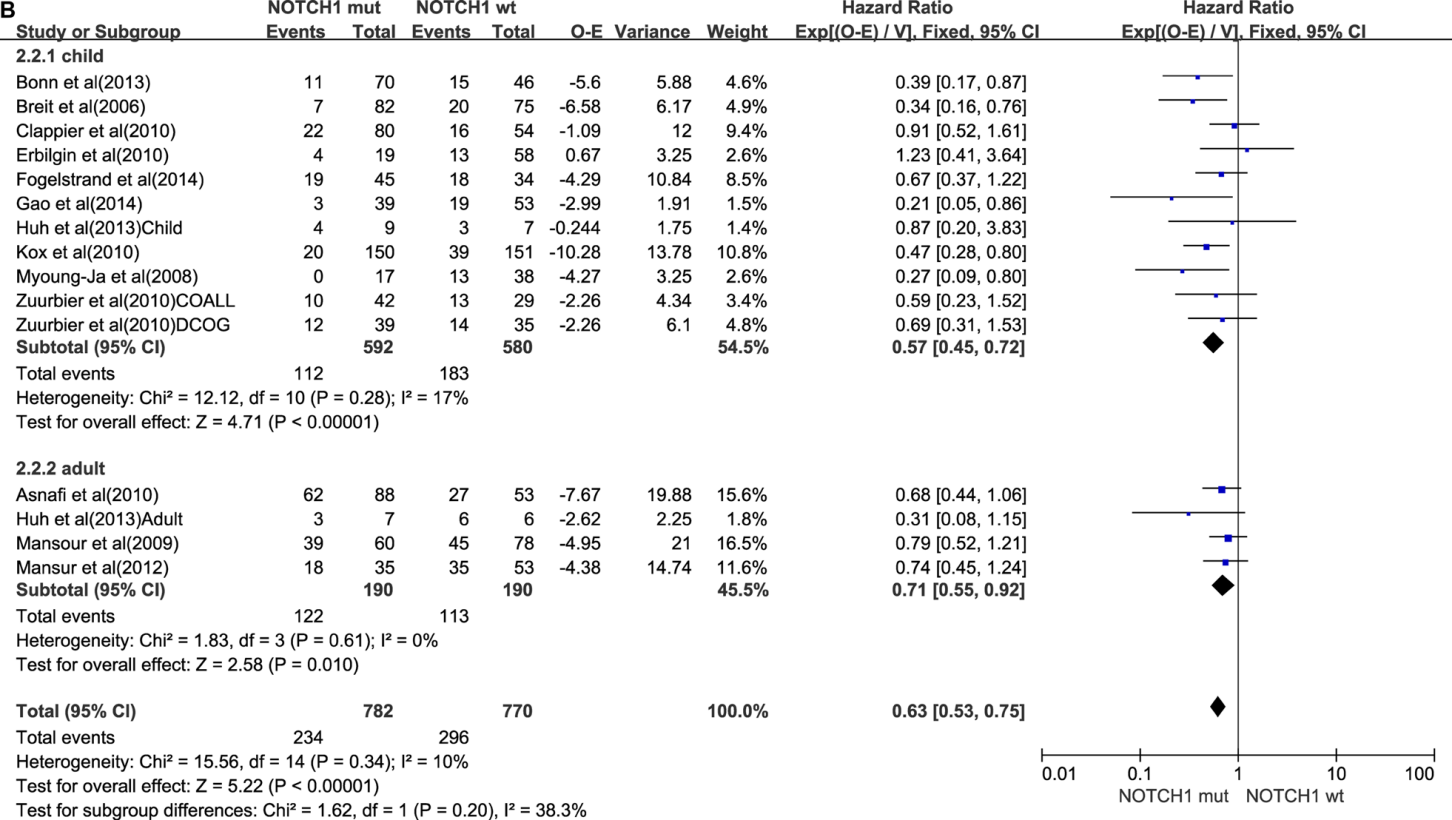
NOTCH1 mutations group (**A**) Forest plot of hazard ratio of 5-year overall survival. (**B**) Forest plot of hazard ratio of 5-year progression-free survival.

A total of 826 T-ALL patients from 16 trials [9–12, 16, 17, 21–27] had NOTCH1-type mutation. Details were shown in Table 2. We divided the 826 patients into a child subgroup and an adult subgroup by the age. Six trials did not provide information about OS. In Callens et al. [28], patients’ total survival periods were up to 4 years. Two trials did not provide patients EFS information. We eliminated those trials. The pooled HRs for 5-year OS and 5-year EFS from all the patients were, respectively, 0.76 (95% CI, 0.56–0.95; *P* = 0.02) and 0.63 (95% CI, 0.53–0.75; *P* < 0.00001). For the child subgroups, relevant HR, 5-year OS, and 5-year EFS were 0.63 (95% CI, 0.42–0.94; *P* = 0.02) and 0.57 (95% CI, 0.45–0.72; *P* < 0.00001). For the adult subgroups, relevant HR, 5-year OS, and 5-year EFS were 0.83 (95% CI, 0.62–1.10; *P* = 0.19) and 0.71 (95% CI, 0.55–0.92; *P* = 0.010). Figure 2A and 2B show that these outcomes are a more favorable survival indication for NOTCH1 mutation–positive patients than for NOTCH1 mutation–negative patients. When compared with adult patients, child patients had a better prognosis.

**Table 2:** Summary of 14 trials of NOTCH1 mutations only included in this meta-analysis

**Source [Reference]**	**Country**	**Age**	**Protocols/Trials**	**Study groups**	**No. of patients**	**No. of events**	
						**OS**	**EFS**
Bonn et al (2013)	USA	Child	NHL-BFM	Mut	70	na	59
				WT	46	na	31
Breit et al (2006)	German	Child	ALL-BFM 2000	Mut	82	na	75
				WT	75	na	55
Clappier et al (2010)	Finland	Child	EORTC58951	Mut	80	63	58
				WT	54	44	38
Erbilgin et al (2010)	Turkey	Child	ALL-BFM protocols	Mut	19	14	15
				WT	68	41	55
Fogelstrand et al (2014)	Sweden	Child	NOPHO ALL-1992 ;1992; ALL-2000	Mut	45	27	26
				WT	34	17	16
Gao et al (2014)	China	Child	BCH-2003 and CCLG-2008	Mut	39	na	36
				WT	53	na	34
Huh et al (2013)	Korean	Child	NA	Mut	9	1	1
				WT	7	0	0
Kox et al (2010)	German	Child	ALL–BFM 2000	Mut	150	na	112
				WT	151	na	130
Myoung-Ja et al (2008)	Japan	Child	(JACLS) protocols ALL-97	Mut	17	11	17
				WT	38	21	23
Zuurbier et al (2010)	German	Child	DCOG protocols	Mut	42	na	32
				WT	29	na	16
Zuurbier et al (2010)	German	Child	COALL protocols	Mut	39	na	25
				WT	35	na	22
Asnafi et al (2010)	France	Adult	LALA-94 ; GRAALL-2003	Mut	88	50	0
				WT	53	26	0
Huh et al (2013)	Korean	Adult	NA	Mut	7	2	2
				WT	6	0	0
Mansour et al (2009)	UK	Adult	UKALLXII/ECOGE2993 protocol	Mut	53	8	8
				WT	35	7	6
Mansur et al (2012)	Brazil	Adult	BFM protocols	Mut	60	32	21
				WT	78	38	33

### FBXW7 mutations

**Figure 3:**
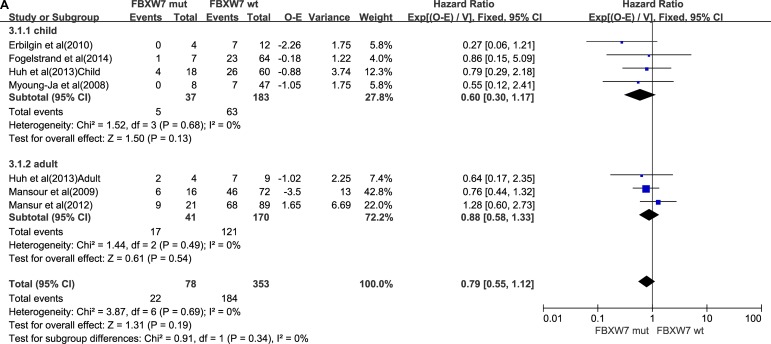

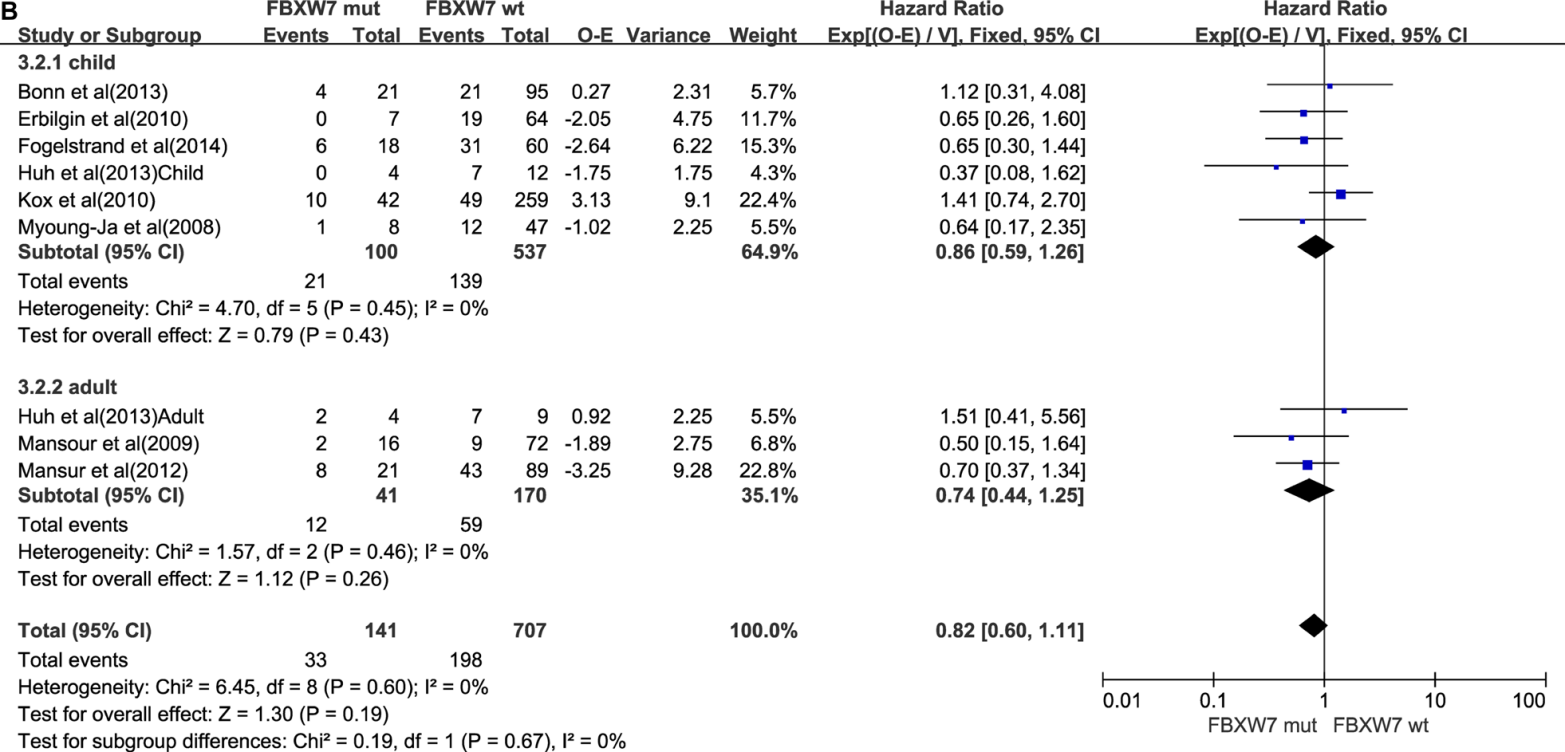
FBXW7 mutations group (**A**) Forest plot of hazard ratio of 5-year overall survival. (**B**) Forest plot of hazard ratio of 5-year progression-free survival.

A total of 197 T-ALL patients in 9 trials [9, 11, 12, 16, 17, 21, 24, 25] harbored only the FBXW7 mutation. Details are shown in Table 3. The 5-year EFS and OS for 10 reports analyzed for FBXW7 mutation status (7 reports for 5-year OS and 9 reports for 5-year EFS). The total-patients group (*n =* 431), had a 5-year OS of HR = 0.79 (FBXW7 mutations status vs wild-type FBXW7, *P* = 0.19). The total-patients group (*n =* 848) had a 5-year EFS of HR = 0.82 (FBXW7 mutations status vs wild-type FBXW7, *P* = 0.19). In the studies of children, the total-patients group (*n =* 220) had a 5-year OS of HR = 0.60 (FBXW7 mutations status vs wild-type FBXW7, *P* = 0.13). The total-patients group (*n =* 637) had a 5-year EFS of HR = 0.86 (FBXW7 mutations status vs wild-type FBXW7, *P* = 0.43). In the adult subgroup, the total-patients group (*n =* 211) had a 5-year OS of HR = 0.88 (FBXW7 mutations status vs wild-type FBXW7, *P* = 0.54). The total-patients group (*n =* 211) had a 5-year EFS of HR = 0.74 (FBXW7 mutations status vs wild-type FBXW7, *P* = 0.26). On the basis of the primary data demonstrated in Figure 3A and 3B

**Table 3:** Summary of nine trials of FBXW7 mutations included in this meta-analysis

Source [Reference]	Country	Age	Protocols/Trials	Study groups	No. of patients	No. of events	
						**OS**	**EFS**
Bonn et al (2013)	USA	Child	NHL-BFM	Mut	21	na	17
				WT	95	na	73
Huh et al (2013)	Korea	Child	NA	Mut	4	1	1
				WT	12	0	0
Kox et al (2010)	Germany	Child	ALL–BFM 2000 study	Mut	42	na	32
				WT	259	na	210
Erbilgin et al (2010)	Turkey	Child	ALL-BFM protocols	Mut	7	6	7
				WT	64	41	45
Fogelstrand et al (2014)	Sweden	Child	NOPHO ALL-1992 ; ALL-2000	Mut	18	14	12
				WT	57	16	15
Myoung-Ja et al (2008)	Japan	Child	(JACLS) protocols ALL-97	Mut	8	8	5
				WT	47	40	22
Huh et al (2013)	Korea	Adult	NA	Mut	4	1	1
				WT	9	1	1
Mansour et al (2009)	U K	Adult	UKALLXII/ECOGE2993	Mut	72	2	2
				WT	16	10	9
Mansur et al (2012)	Brazil	Adult	BFM protocols	Mut	21	13	12
				WT	89	46	37

We concluded that the FBXW7 mutation did not have a significant effect on the prognosis of the child patients and the adult patients.

In general, concurrent NOTCH1 and FBXW7 mutations were associated with a better patient survival than the NOTCH1 mutations.

### Heterogeneity analysis and publication

Heterogeneity, which correlated with the pooled OS and EFS HRs, was not distinct because the values were all less than 50%. Six funnel plots were almost symmetrical, indicating no obvious publication bias existed in our meta-analysis (Supplementary Figure 2).

## DISCUSSION

Ma et al. [29] carried out a literature-based meta-analysis that focused on the effect of NOTCH1 expression for the prognosis of T-ALL and found no beneficial effects on EFS in the NOTCH1 mutations subgroup. However, these original results are incomplete and the lack of a systematic review evaluation failed to give further insights on this issue. In our meta-analysis, we found that T-ALL patients with NOTCH1 mutations alone or in combination with FBXW7 have a better prognosis compared with those without FBXW7 mutations, without NOTCH1 mutations, or both, especially in the NOTCH1/FBXW7 subgroup. The statistically significant association with 5-year OS and EFS was detected in our study. We found that NOTCH1/FBXW7 mutations significantly increased the risk of long-term prognosis. Furthermore, the HR for 5-year EFS and OS in patients with NOTCH1 mutations were 0.63 (95% CI, 0.53 to 0.75) and 0.76 (95% CI, 0.60 to 0.95), and the HR for 5-year EFS and OS in patients with FBXW7 mutations were 0. 82 (95% CI, 0.60 to 1.11) and 0.79 (95% CI, 0.55 to 1.12). In all T-ALL patients in the 18 trials, we determined the incidence of activating mutations. The incidence in NOTCH1/FBXW7 genes was 60.60%. In addition, the number in NOTCH1 and FBXW7 genes was 51.11% and 22.38%. When we studied the child and adult subgroups separately, we found that the mutation event rate was a little higher in the adult subgroup.

Previous research showed the prognostic effect of the NOTCH1 mutation was linked to the ages of the patients [30]. Because child patients respond well to current therapy, the significance of molecular biomarkers can be decreased by the relatively good prognosis in the child subgroup, and no significant superiority of survival time was observed in patients without NOTCH1 mutations. We conducted the stratified analysis by age to determine the correlation between NOTCH1 and FBXW7 gene mutations and T-ALL prognosis. In the subgroup analysis according to age, for 5-year EFS, no significant associations were found among adult and child subgroups. Evidence indicates that children with mutations status have better 5-year OS than adults . However, both in child and in adult groups, the NOTCH1/FBXW7 mutations were correlated with a better 5-year EFS and 5-year OS. The presence of NOTCH1/FBXW7 mutations is a positive prognostic factor for T-ALL patient survival.

In the present meta-analysis, between-study heterogeneities for the overall data were absent in the six comparisons. Thus, the fixed-effect models were utilized. In the subgroup analyses, insignificant heterogeneities were also found in the relevant subgroups, increasing the stability and reliability of the results [31]. Several limitations should be addressed. First, the number of articles included was relatively small, especially for the FBXW7 group. Second, subgroup analyses with respect to treatment regimen, test methods, and other factors were not conducted in the present study because of the lack of relevant data in the primary literature. Third, some heterogeneity exists in the relation between mutations status and cancer prognosis. When the Calles article [28] was excluded, the heterogeneity disappeared. Despite the limitations, our meta-analysis indicated that T-ALL patients with concurrent NOTCH1 and FBXW7 mutations or a single NOTCH1 mutation had a significantly better survival than patients with wild-type FBXW7. So detecting mutation status by adopting molecular diagnosis could be an appropriate consideration. This method could be a beneficial therapeutic strategy.

## MATERIALS AND METHODS

### Literature search strategy and study criterion

A sensitive search strategy was developed for all English language literature published before May 2014. The comprehensive search was performed by use of the electronic databases PubMed, EMBASE, and the Cochrane Library. The search terms were T-cell acute lymphoblastic leukemia or T-ALL, NOTCH1, and FBXW7. The search was limited to randomized controlled trials or clinical trials. Meta-analysis articles were not excluded because several original studies were often combined in meta-analysis articles.

The selection criteria were as follows:

(a) All T-ALL patients were diagnosed by immunophenotypic analyses according to World Health Organization classification. (b) NOTCH1 and FBXW7 mutation were identified by direct sequencing of polymerase chain reaction–amplified products. (c) Case studies, review articles, and studies involving fewer than three patients were excluded. (d) Randomized controlled trials compared immunotherapy versus control therapy and included children and adults. (e) Adequate survival information and patterns of failure data were needed.

### Data extraction and quality control

Two reviewers (RB. L and TZ. L) performed data extraction independently by use of standardized data extraction sheets. These data included study design, year of publication, population characteristics (country of patients and age), follow-up duration, trials, study groups, number of patients in each group and number of events in each group. Survival estimates were extracted directly from the text or deduced from the survival curve of the publication [32]. The principal summary measure was the hazard ratios (HRs), which could be extracted from the text or deduced from the original data. If the article did not provide the HRs for OS or event-free survival EFS, the Engauge Digitizer Version 4.1 software was used to distinguish the survival curves and calculate the HRs of OS and EFS. We extracted the *P* value of the log-rank test of the 5-year OS or the 5-year EFS.

To assign study quality, we examined the randomization procedure, estimation of sample size, allocation of concealment blinding of outcome assessor, loss to follow-up, dropout, and whether the intention-to-treat analysis was followed. The Jadad/Oxford quality scoring system was used to quantify study quality [33]. The study quality is shown in Supplementary Table 1. Any discrepancies in abstracting data were resolved by consensus.

### Data synthesis and analysis

Considering that NOTCH1 and FBXW7 belong to the same signaling pathway, the trials were classified into three subgroups, NOTCH1 alone; FBXW7 alone; and NOTCH1, FBXW7, or both, based on the mutations status of T-ALL patients. Subgroup analyses of the end events were performed by utilization of HR to assess the differences between the mutations arm and wild-type arm in each subgroup. Moreover, in each group, children and adults were analyzed. The main endpoint was OS, defined as the period from the randomization date to the date of death. Secondary endpoints were PFS, defined as the period from the randomization date to the date when disease progression (or death) was observed. Results regarding OS were expressed as HR with 95% CI, because it was the only summary statistic that allowed for both censoring and time to an event [34, 35]. The HRs and 95% CIs for the available data were calculated to identify potential correlations with overall survival in the two groups by use of the method reported by Tierney et al. [36]. An HR < 1 means a lower rate of events in the mutations arm.

Statistical analysis was carried out by Review Manager Version 5.3 software provided by the Cochrane Collaboration. Heterogeneity was calculated by application of the Q-statistic and the I2 statistic. If the *Q* statistic *P* value > 0.1, the HRs were pooled according to a fixed-effect model (Mantel-Haenszel). Otherwise, a random-effect model (DerSimonian and Laird) was selected. For I2, values of 25% to < 50% were considered small, 50% to < 75% were considered medium, and ≥ 75% were considered large [37].

### Recognition of relevant researches

A total of 645 reports were retrieved when the electronic screening job was finished. After abstract review, 615 articles were excluded and 28 full-text articles remained. Afterward, 10 articles were eliminated for the following reasons: Six articles did not include NOTCH1 or FBXW7 mutation in their studies. Two articles did not analyze OS or EFS. Two articles were about the same trails in different publications. Eventually, 18 studies were eligible. The flow is shown in Supplementary Figure 1.

## SUPPLEMENTARY MATERIALS FIGURES AND TABLE


