# Supplementary material for: Meta-analysis of the clinical characteristics and prognostic relevance of NOTCH1 and FBXW7 mutation in T-cell acute lymphoblastic leukemia
Source: Oncotarget. 2017 Jun 19;8(39):66360–70. doi: 10.18632/oncotarget.18576 (PMC5630418; doi:10.18632/oncotarget.18576)
Supplement: Supplementary file 1 [file oncotarget-08-66360-s001.pdf]

Meta-analysis of the clinical characteristics and prognostic relevance of NOTCH1 and FBXW7 mutation in T-cell acute lymphoblastic leukemia

Supplementary Materials

Study criteria:

(a) All T-ALL patients diagnosed by immunophenotypic analyses according to WHO classification;
(b)NOTCH1 and FBXW7 mutation identified by direct sequencing of polymerase chain reaction (PCR)-amplified products;
(c) Case studies, review articles, and studies involving fewer than three patients were excluded;
(d) Randomized controlled trials to compare immunotherapy versus control therapy and including children and adults;
(e) Adequate survival information and patterns of failure data were needed.

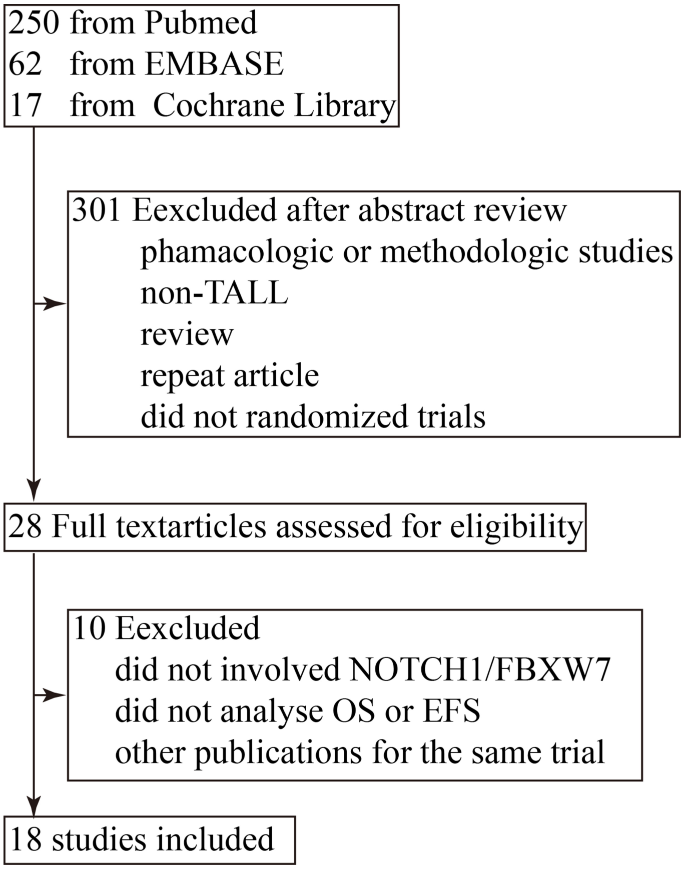

Supplementary Figure 1: Flow of identifying the 18 trials.

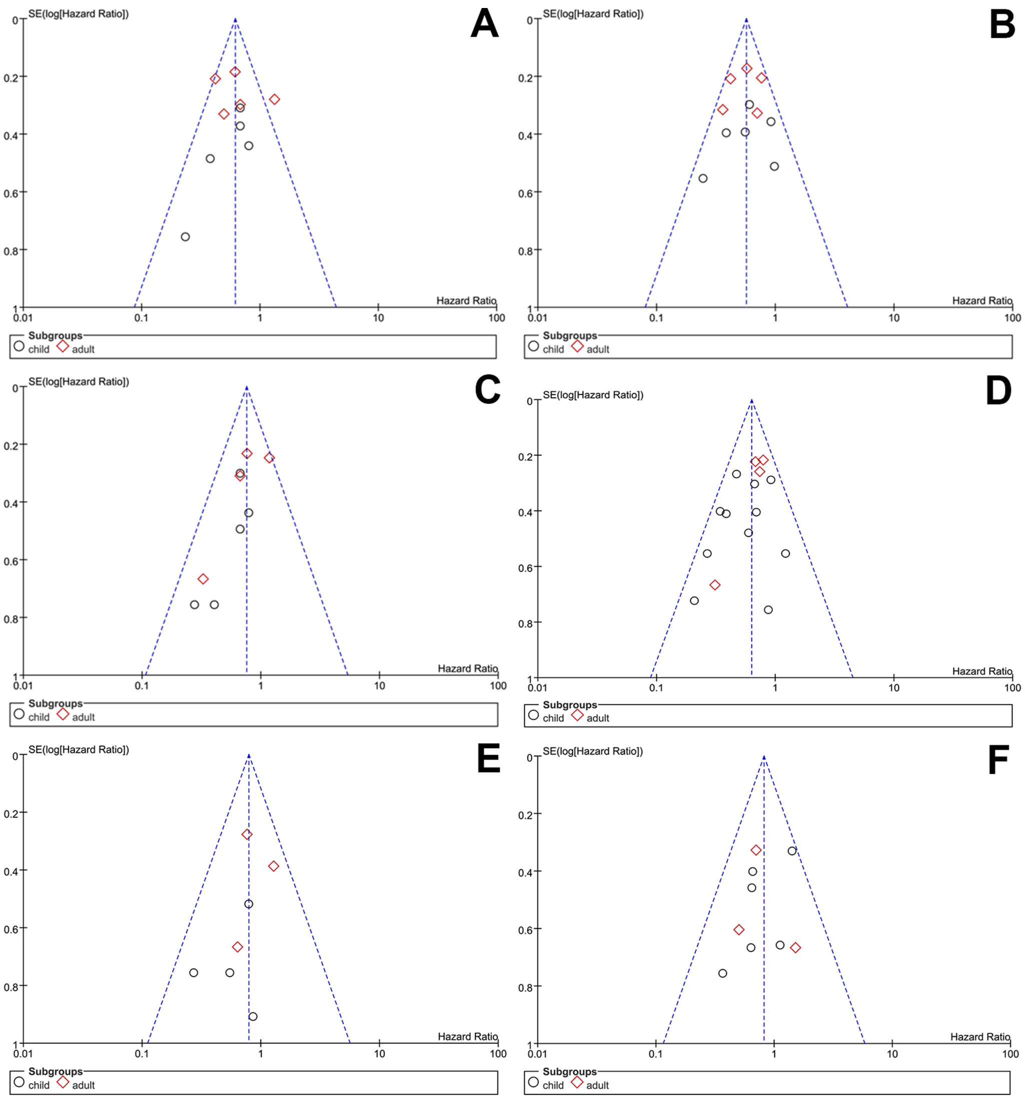

Supplementary Figure 2: Six funnel plots in this meta-analysis. (**A**) funnel plot of hazard ratio (HR) of 5-year overall survival in NOTCH1/FBXW7 mutations group. (**B**) Funnel plot of hazard ratio (HR) of 5-year progression-free survival in NOTCH1/FBXW7 mutations group. (**C**) Funnel plot of hazard ratio (HR) of 5-year overall survival in NOTCH1 mutations group. (**D**) Funnel plot of hazard ratio (HR) of 5-year progression-free survival in NOTCH1 mutations group. (**E**) Funnel plot of hazard ratio (HR) of 5-year overall survival in FBXW7 mutations group. (**F**) Funnel plot of hazard ratio (HR) of 5-year progression-free survival in FBXW7 mutations group.

Supplementary Table 1: Assessing quality of all the included studies

Studies	Randomization procedure	Estimation of sample size	Allocation concealment blinding of outcome assessor	Loss to follow-up	dropout	Jadad scores
Abdelali et al. (2011)	Yes	Yes	No	Yes	Yes	3
Asnafi et al. (2010)	Yes	Yes	No	Yes	Yes	3
Baldus et al. (2009)	Yes	Yes	No	No	Yes	3
Breit et al. (2006)	Yes	Yes	No	Yes	Yes	3
Bonn et al. (2013)	Yes	Yes	No	Yes	Yes	3
Callens et al. (2012)	Yes	Yes	No	Yes	Yes	3
Clappier et al. (2010)	Yes	Yes	No	No	Yes	3
Erbilgin et al. (2010)	Yes	Yes	No	No	Yes	3
Fogelstrand et al. (2014)	Yes	Yes	No	Yes	Yes	3
Gao et al. (2014)	Yes	Yes	No	Yes	Yes	3
Huh et al. (2013)	Yes	Yes	No	Yes	Yes	3
Kox et al. (2010)	Yes	Yes	No	Yes	Yes	3
Jenkinson et al. (2010)	Yes	Yes	No	Yes	Yes	3
Mansour et al. (2009)	Yes	Yes	No	Yes	Yes	3
Mansur et al. (2012)	No	No	No	Yes	Yes	2
Myoung-Ja et al. (2008)	No	No	No	Yes	Yes	2
Trinquand et al. (2013)	Yes	Yes	No	Yes	Yes	3
Zuurbier et al. (2010)	Yes	Yes	No	Yes	Yes	3

REFERENCES

1. Ben Abdelali R, Asnafi V, Leguay T, Boissel N, Buzyn A, Chevallier P, Thomas X, Lepretre S, Huguet F, Vey N, Escoffre-Barbe M, Tavernier E, Reman O, et al. Pediatric-inspired intensified therapy of adult T-ALL reveals the favorable outcome of NOTCH1/FBXW7 mutations, but not of low ERG/BAALC expression: a GRAALL study. Blood. 2011; 19:5099–5107.
2. Asnafi V, Buzyn A, Le Noir S, Baleydier F, Simon A, Beldjord K, Reman O, Witz F, Fagot T, Tavernier E, Turlure P, Leguay T, Huguet F, et al. NOTCH1/FBXW7 mutation identifies a large subgroup with favorable outcome in adult T-cell acute lymphoblastic leukemia (T-ALL): a Group for Research on Adult Acute Lymphoblastic Leukemia (GRAALL) study. Blood. 2009; 17:3918–3924.
3. Baldus CD, Thibaut J, Goekbuget N, Stroux A, Schlee C, Mossner M, Burmeister T, Schwartz S, Bloomfield CD, Hoelzer D, Thiel E, Hofmann WK. Prognostic implications of NOTCH1 and FBXW7 mutations in adult acute T-lymphoblastic leukemia. Haematologica. 2009; 10: 1383–1390.
4. Breit S, Stanulla M, Flohr T, Schrappe M, Ludwig WD, Tolle G, Happich M, Muckenthaler MU, Kulozik AE. Activating NOTCH1 mutations predict favorable early treatment response and long-term outcome in childhood precursor T-cell lymphoblastic leukemia. Blood. 2006; 4:1151–1157.
5. Bonn BR, Rohde M, Zimmermann M, Krieger D, Oschlies I, Niggli F, Wrobel G, Attarbaschi A, Escherich G, Klapper W, Reiter A, Burkhardt B. Incidence and prognostic relevance of genetic variations in T-cell lymphoblastic lymphoma in childhood and adolescence. Blood. 2013; 16:3153–3160.
6. Callens C, Baleydier F, Lengline E, Ben Abdelali R, Petit A, Villarese P, Cieslak A, Minard-Colin V, Rullier A, Moreau A, Baruchel A, Schmitt C, Asnafi V, et al. Clinical impact of NOTCH1 and/or FBXW7 mutations, FLASH deletion, and TCR status in pediatric T-cell lymphoblastic lymphoma. J Clin Oncol. 2012; 16:1966–1973.
7. Clappier E, Collette S, Grardel N, Girard S, Suarez L, Brunie G, Kaltenbach S, Yakouben K, Mazingue F, Robert A, Boutard P, Plantaz D, Rohrlich P, et al. NOTCH1 and FBXW7 mutations have a favorable impact on early response to treatment, but not on outcome, in children with T-cell acute lymphoblastic leukemia (T-ALL) treated on EORTC trials 58881 and 58951. Leukemia. 2010; 12:2023–2031.
8. Erbilgin Y, Sayitoglu M, Hatirnaz O, Dogru O, Akcay A, Tuysuz G, Celkan T, Aydogan G, Salcioglu Z, Timur C, Yuksel-Soycan L, Ure U, Anak S, et al. Prognostic significance of NOTCH1 and FBXW7 mutations in pediatric T-ALL. Dis Markers. 2010; 6:353–360.

9. Fogelstrand L, Staffas A, Wasslavik C, Sjogren H, Soderhall S, Frost BM, Forestier E, Degerman S, Behrendtz M, Heldrup J, Karrman K, Johansson B, Heyman M, et al. Prognostic implications of mutations in NOTCH1 and FBXW7 in childhood T-ALL treated according to the NOPHO ALL-1992 and ALL-2000 protocols. Pediatr Blood Cancer. 2014; 3:424–430.
10. Gao C, Liu SG, Zhang RD, Li WJ, Zhao XX, Cui L, Wu MY, Zheng HY, Li ZG. NOTCH1 mutations are associated with favourable long-term prognosis in paediatric T-cell acute lymphoblastic leukaemia: a retrospective study of patients treated on BCH-2003 and CCLG-2008 protocol in China. Br J Haematol. 2014; 2:221–228.
11. Huh HJ, Lee SH, Yoo KH, Sung KW, Koo HH, Jang JH, Kim K, Kim SJ, Kim WS, Jung CW, Lee KO, Kim SH, Kim HJ. Gene mutation profiles and prognostic implications in Korean patients with T-lymphoblastic leukemia. Ann Hematol. 2013; 5:635–644.
12. Kox C, Zimmermann M, Stanulla M, Leible S, Schrappe M, Ludwig WD, Koehler R, Tolle G, Bandapalli OR, Breit S, Muckenthaler MU, Kulozik AE. The favorable effect of activating NOTCH1 receptor mutations on long-term outcome in T-ALL patients treated on the ALL-BFM 2000 protocol can be separated from FBXW7 loss of function. Leukemia. 2010; 12:2005–2013.
13. Jenkinson S, Koo K, Mansour MR, Goulden N, Vora A, Mitchell C, Wade R, Richards S, Hancock J, Moorman AV, Linch DC, Gale RE. Impact of NOTCH1/FBXW7 mutations on outcome in pediatric T-cell acute lymphoblastic leukemia patients treated on the MRC UKALL 2003 trial. Leukemia. 2013; 1:41–47.
14. Mansour MR, Sulis ML, Duke V, Foroni L, Jenkinson S, Koo K, Allen CG, Gale RE, Buck G, Richards S, Paietta E, Rowe JM, Tallman MS, et al. Prognostic implications of NOTCH1 and FBXW7 mutations in adults with T-cell acute lymphoblastic leukemia treated on the MRC UKALLXII/ ECOG E2993 protocol. J Clin Oncol. 2009; 26:4352–4356.
15. Mansur MB, Hassan R, Barbosa TC, Splendore A, Jotta PY, Yunes JA, Wiemels JL, Pombo-de-Oliveira MS. Impact of complex NOTCH1 mutations on survival in paediatric T-cell leukaemia. BMC Cancer. 2012:9.
16. Park MJ, Taki T, Oda M, Watanabe T, Yumura-Yagi K, Kobayashi R, Suzuki N, Hara J, Horibe K, Hayashi Y. FBXW7 and NOTCH1 mutations in childhood T cell acute lymphoblastic leukaemia and T cell non-Hodgkin lymphoma. Br J Haematol. 2009; 2:198–206.
17. Trinquand A, Tanguy-Schmidt A, Ben Abdelali R, Lambert J, Beldjord K, Lengline E, De Gunzburg N, Payet-Bornet D, Lhermitte L, Mossafa H, Lheritier V, Bond J, Huguet F, et al. Toward a NOTCH1/FBXW7/RAS/PTEN-based oncogenetic risk classification of adult T-cell acute lymphoblastic leukemia: a Group for Research in Adult Acute Lymphoblastic Leukemia study. J Clin Oncol. 2013; 34:4333–4342.
18. Zuurbier L, Homminga I, Calvert V, te Winkel ML, Buijs-Gladdines JG, Kooi C, Smits WK, Sonneveld E, Veerman AJ, Kamps WA, Horstmann M, Petricoin EF 3rd, et al. NOTCH1 and/or FBXW7 mutations predict for initial good prednisone response but not for improved outcome in pediatric T-cell acute lymphoblastic leukemia patients treated on DCOG or COALL protocols. Leukemia. 2010; 12:2014–2022.
